# Trends in prevalence and sociodemographic and geographic patterns of current menthol cigarette use among U.S. adults, 2005–2015

**DOI:** 10.1016/j.pmedr.2020.101227

**Published:** 2020-11-10

**Authors:** Delvon T. Mattingly, Jana L. Hirschtick, Rafael Meza, Nancy L. Fleischer

**Affiliations:** University of Michigan School of Public Health, Department of Epidemiology, Ann Arbor, MI 48109, USA

**Keywords:** Smokers, Menthol, Cigarette smoking, Tobacco products, Epidemiology, Public health, Health status disparities

## Abstract

Despite overall reductions in U.S. smoking prevalence, prior evidence suggests similar reductions may not have occurred for menthol cigarette users. This study examines nationally representative current menthol and non-menthol cigarette use prevalence and trends for adults (18+) overall and by sociodemographic and geographic characteristics using the 2005 (n = 31,132), 2010 (n = 26,967), and 2015 (n = 33,541) National Health Interview Survey. Between 2005 and 2015, non-menthol cigarette use decreased overall (14.7% to 9.6%, p < 0.001) and within all sociodemographic and geographic subgroups analyzed (i.e., by sex, age, race/ethnicity, sexual orientation, education, family income, and geographic region), except non-Hispanic American Indians/Alaskan Natives (NH AI/AN) and non-Hispanic Others. Menthol cigarette use significantly decreased overall (5.3% to 4.4%, p < 0.001), and among females (5.6% to 4.6%); participants aged 18–24 (7.1% to 4.3%) and 35–54 (6.2% to 4.9%); non-Hispanic Whites (4.1% to 3.6%) and non-Hispanic Blacks (14.8% to 11.9%); participants with high school degrees/GEDs (7.0% to 5.9%); participants with a family income of $75,000 or higher (3.4% to 2.3%); and participants residing in the Northeast (6.0% to 4.3%). Menthol cigarette use remained stable or did not significantly decrease among males; adults aged 25–34 and 55 years and older; NH AI/ANs, NH Others, and Hispanics; participants with less than high school education, some college, or a college degree; participants with a family income below $75,000; and participants residing in the North Central/Midwest, South, and West. Given that menthol cigarette use did not significantly change or decrease for multiple subgroups, further restriction on menthol manufacturing may help reduce tobacco use disparities.

## 1 Introduction

Approximately 40% of US smokers prefer menthol rather than non-menthol cigarettes (Villanti et al., 2016). Although the prevalence of non-menthol cigarette use is decreasing, the prevalence of menthol cigarette use has either increased or remained unchanged in certain sociodemographic groups (Curtin et al., 2014, Giovino et al., 2015, Villanti et al., 2016). Data from the National Survey of Drug Use and Health (NSDUH) shows that the prevalence of menthol cigarette use stayed constant from 2004 to 2010 among adults aged 26 + years but increased among adults aged 18–25 from 14.0% to 16.3% (Giovino et al., 2015). Among past-30 day cigarette smokers, the proportion of menthol cigarette use increased between 2008 and 2014 overall (34.7% to 38.8%), in males (30.9% to 34.8%), females (39.1% to 43.5%), Hispanics (37.1% to 46.9%), non-Hispanic (NH) Whites (25.6% to 28.9%), NH Asians (30.3% to 38.0%), and individuals with <$30,000 (38.6% to 43.7%) or between $30,000-$74,999 (33.2% to 37.2%) family income (Villanti et al., 2016). These results suggest that efforts to curtail menthol cigarette use, including a federal menthol ban, could have a positive impact on menthol-related smoking disparities.

Menthol cigarette use is most prevalent among NH Black and low socioeconomic status (SES) populations and may lead to worse health outcomes among these groups (Giovino et al., 2015, Villanti et al., 2016). For example, young adult menthol cigarette users have a higher risk of nicotine dependence than non-menthol users (Fagan et al., 2015). Menthol cigarette users are also less successful in quitting than non-menthol cigarette users, possibly due to persistent targeting by the tobacco industry or the perception that menthol is less harmful, with success varying by race/ethnicity (Gundersen et al., 2009, Villanti et al., 2017, Weinberger et al., 2019). Thus, further investigation on the patterning of prevalence of menthol and non-menthol cigarette use, including differences in use over time, is warranted.

We build on prior work that used NSDUH data to compare menthol and non-menthol cigarette use prevalence by examining data from the 2005–2015 National Health Interview Survey (NHIS). We also characterize differences in time trends of menthol and non-menthol cigarette use by sex, age, race/ethnicity, sexual orientation, education, family income, and geographic region in the United States.

## 2 Materials and methods

### 2.1 Design

We analyzed data on adults aged 18 years and older from the 2005 (n = 31,132), 2010 (n = 26,967), and 2015 (n = 33,541) NHIS available from the Integrated Public Use Microdata Series (Lynn et al., 2019). NHIS is a cross-sectional, nationally representative study conducted annually by the National Center for Health Statistics. In 2005, 2010, and 2015, NHIS Cancer Control Supplements included questions on cigarette brand preference, which allows for the identification of menthol smokers (National Center for Health Statistics, 2016).

### 2.2 Measures

#### 2.2.1 Smoking status

Current cigarette users were participants who had smoked 100 cigarettes in their lifetime and indicated they now smoke cigarettes every day or some days. Menthol cigarette users were current cigarette users who indicated menthol as their usual brand, while non-menthol cigarette users were current cigarette users who indicated plain or no preference as their usual brand. Current cigarette users without information on brand preference (i.e., non-classifiable) (n = 945), including those who refused to answer (n = 103), did not have a usual brand preference (n = 806), or did not know their brand preference (n = 36), were classified separately and not included in menthol and non-menthol cigarette use groups. Tobacco use was categorized as current menthol cigarette use, current non-menthol cigarette use, and former or never cigarette use (i.e., non-current cigarette use). Like non-classifiable users, participants missing values for cigarette use (n = 617) were excluded from this analysis.

#### 2.2.2 Sociodemographic Characteristics

Sex was dichotomized as male or female. Age was categorized as 18–24, 25–34, 35–54, and 55 years or older. Race/ethnicity was defined as NH White, NH Black, NH Asian, NH American Indian/Alaskan Native (AI/AN), NH Other, and Hispanic. Sexual orientation (only available in 2015) was categorized as heterosexual versus lesbian, gay, or bisexual. Categorical educational attainment was restricted to respondents aged 25 years and older: less than high school degree, high school degree or GED, some college, and college degree. Total combined family income was categorized as <$35,000, $35,000-$74,999, and $75,000 or more. Geographic region included Northeast, North Central/Midwest, South, and West categories (U.S. Bureau of the Census, 1994).

### 2.3 Statistical analyses

All analyses were weighted to account for the complex survey design of NHIS and conducted using Stata 15.1 (StataCorp, 2017). Weighted prevalence estimates and 95% confidence intervals for current menthol and non-menthol use were computed overall and by sex, age, race/ethnicity, sexual orientation, education, family income, and geographic region. Percent change and tests for differences in proportions using Stata’s linear combinations of estimates (lincom) command were calculated to examine changes in prevalence of current menthol and non-menthol cigarette use from 2005 to 2010, 2010–2015, and 2005–2015.

## 3 Results

Overall, the prevalence of current cigarette use was 20.9% in 2005, 19.3% in 2010, and 15.1% in 2015 (Table A). The prevalence of menthol cigarette use was 5.3%, 5.7%, and 4.4% in 2005, 2010, and 2015, respectively, while the prevalence of non-menthol cigarette use was 14.7%, 12.4%, and 9.6% in 2005, 2010, and 2015, respectively (Table 1). In 2015, the prevalence of menthol cigarette use was similar between women (4.6%) and men (4.3%), but higher for participants aged 25–34 (6.6%) than other age groups; NH Blacks (11.9%) than other racial/ethnic groups; participants with less than a high school degree (6.6%), a high school degree/GED (5.9%), and some college (5.2%) compared to a college degree; participants with a family income of less than $35,000 (7.0%) than those with a higher income level; and participants residing in the North Central/Midwest (5.3%), South (5.2%), and Northeast (4.3%), compared to the West. In 2015, compared to heterosexual participants, sexual minority participants had higher prevalence of menthol cigarette use (8.0%) and non-menthol cigarette use (12.3%).

**Table 1:** Prevalence of Current Menthol and Non-Menthol Cigarette Use by Sociodemographic and Geographic Characteristics, 2005–2015.

Characteristics	Menthol						Non-Menthol							
	*n*	2005% (95% CI)	*n*	2010% (95% CI)	*n*	2015% (95% CI)	*n*	2005% (95% CI)		*n*	2010% (95% CI)	*n*	2015% (95% CI)	
Overall	1700	5.3 (4.9, 5.6)	1689	5.7 (5.4, 6.1)	1616	4.4 (4.2, 4.7)	4499	14.7	(14.2, 15.2)	3157	12.4 (11.9, 12.9)	3467	9.6	(9.1, 10.1)
Sex														
Male	684	4.9 (4.4, 5.3)	748	5.7 (5.3, 6.2)	686	4.3 (3.9, 4.7)	2478	17.9	(17.1, 18.8)	1709	14.5 (13.7, 15.2)	1895	11.3 (10.6, 12.2)	
Female	1016	5.6 (5.2, 6.1)	941	5.8 (5.3, 6.2)	930	4.6 (4.2, 5.0)	2021	11.7	(11.1, 12.3)	1448	10.5 (9.9, 11.1)	1572	8.0 (7.5, 8.5)	

Age group														
18–24	229	7.1 (6.1, 8.4)	233	8.4 (7.3, 9.8)	152	4.3 (3.4, 5.4)	499	16.2	(14.4, 18.1)	289	10.8 (9.4, 12.4)	236	7.8	(6.5, 9.3)
25–34	350	5.7 (4.9, 6.5)	414	8.2 (7.4, 9.2)	391	6.6 (5.8, 7.6)	953	17.7	(16.5, 19.0)	626	13.7 (12.6, 14.9)	614	9.9	(8.8, 11.0)
35–54	784	6.2 (5.7, 6.8)	647	5.6 (5.1, 6.2)	587	4.9 (4.4, 5.5)	1984	16.6	(15.8, 17.5)	1342	14.8 (13.9, 15.8)	1409	11.8	(11.0, 12.7)
55+	337	2.9 (2.6, 3.3)	395	3.5 (3.1, 3.9)	486	2.9 (2.6, 3.3)	1063	9.7	(9.1, 10.4)	900	9.6 (8.9, 10.3)	1208	8.0	(7.4, 8.7)

Race/ethnicity														
Non-Hispanic White	827	4.1 (3.8, 4.4)	739	4.7 (4.3, 5.1)	751	3.6 (3.2, 3.9)	3433	16.9	(16.2, 17.6)	2368	15.1 (14.4, 15.8)	2702	11.9	(11.3, 12.6)
Non-Hispanic Black	609	14.8 (13.4, 16.3)	668	14.8 (13.5, 16.2)	564	11.9 (10.6, 13.2)	266	5.1	(4.5, 5.9)	206	4.3 (3.6, 5.1)	179	3.4	(2.8, 4.3)
Non-Hispanic Asian	32	4.2 (2.8, 6.1)	49	3.0 (2.2, 4.1)	42	1.7 (1.1, 2.5)	87	8.8	(6.7, 11.5)	120	5.9 (4.8, 7.3)	109	4.9	(3.8, 6.4)
Non-Hispanic AI/AN	10	4.8 (2.2, 10.0)	10	8.1 (3.6, 17.4)	27	4.7 (2.6, 8.5)	40	27.2	(19.8, 36.1)	41	27.0 (18.6, 37.5)	61	17.0	(11.1, 25.0)
Non-Hispanic Other	9	14.3 (8.3, 23.7)	13	13.9 (6.7, 26.6)	14	7.2 (3.2, 15.3)	6	6.1	(2.5, 14.3)	7	7.0 (3.3, 14.1)	17	5.8	(3.1, 10.7)
Hispanic	213	3.7 (3.0, 4.5)	210	3.9 (3.3, 4.6)	218	3.3 (2.8, 3.9)	667	11.9	(10.9, 13.0)	415	7.9 (7.1, 8.9)	399	6.1	(5.4, 7.0)

Sexual orientation														
Straight/heterosexual	–	–	–	–	1517	4.5 (4.2, 4.8)	–		–	–	–	3301	9.8	(9.3, 10.4)
Lesbian/gay/bisexual	–	–	–	–	72	8.0 (5.9, 10.7)	–		–	–	–	119	12.3	(9.7, 15.6)

Education														
Less then high school	397	7.2 (6.4, 8.2)	381	8.3 (7.4, 9.3)	306	6.6 (5.8, 7.6)	934	18.4	(16.9, 20.0)	614	16.2 (15.0, 17.6)	658	15.0	(13.6, 16.4)
High school degree/GED	611	7.0 (6.3, 7.6)	583	7.9 (7.2, 8.6)	548	5.9 (5.2, 6.6)	1569	18.5	(17.6, 19.5)	1059	16.3 (15.2, 17.5)	1166	13.4	(12.3, 14.5)
Some college	521	5.6 (5.1, 6.2)	549	6.0 (5.4, 6.7)	584	5.2 (4.6, 5.8)	1352	15.4	(14.6, 16.3)	1024	13.1 (12.1, 14.1)	1165	10.1	(9.3, 10.9)
College degree	162	1.8 (1.5, 2.1)	168	2.1 (1.8, 2.5)	170	1.6 (1.3, 1.9)	611	7.3	(6.7, 7.9)	452	6.1 (5.4, 6.8)	461	4.0	(3.5, 4.5)

Family income														
Less than $35,000	1007	6.8 (6.3, 7.3)	1043	8.7 (8.1, 9.4)	939	7.0 (6.4, 7.7)	2387	17.3	(16.3, 18.2)	1622	15.5 (14.7, 16.5)	1875	13.8	(12.9, 14.7)
$35,000-$74,999	474	5.2 (4.6, 5.8)	436	5.2 (4.7, 5.8)	461	4.6 (4.1, 5.2)	1430	15.6	(14.7, 16.5)	1014	13.3 (12.5, 14.2)	1003	10.3	(9.5, 11.2)
$75,000+	219	3.4 (2.9, 4.0)	210	3.2 (2.8, 3.8)	216	2.3 (1.9, 2.7)	682	10.3	(9.6, 11.2)	521	8.2 (7.5, 9.0)	589	5.8	(5.2, 6.5)

Geographic region														
Northeast	369	6.0 (5.3, 6.8)	279	6.0 (5.3, 6.9)	270	4.3 (3.7, 5.2)	639	12.0	(10.9, 13.2)	436	10.2 (9.1, 11.5)	486	7.9	(6.8, 9.1)
North central/midwest	480	6.1 (5.4, 6.8)	442	6.4 (5.7, 7.2)	364	5.3 (4.6, 6.1)	1200	17.2	(16.1, 18.2)	796	14.0 (12.9, 15.2)	857	12.0	(10.8, 13.3)
South	663	5.9 (5.3, 6.6)	736	6.8 (6.3, 7.4)	698	5.2 (4.8, 5.7)	1672	14.9	(13.9, 15.9)	1153	12.8 (12.0, 13.7)	1116	9.1	(8.4, 9.9)
West	188	2.6 (2.1, 3.1)	232	3.2 (2.8, 3.8)	284	2.4 (2.0, 2.8)	988	13.8	(12.8, 14.8)	772	11.8 (10.8, 13.0)	1008	9.4	(8.5, 10.4)

628 participants were missing values for education.

1,397 participants were missing values for sexual orientation in NHIS 2015.

Sources: National Health Interview Survey, 2005, 2010, and 2015.

NHIS 2005 sample size = 31,132; NHIS 2010 sample size = 26,967; NHIS 2015 sample size = 33,541.

**Fig. 1:**
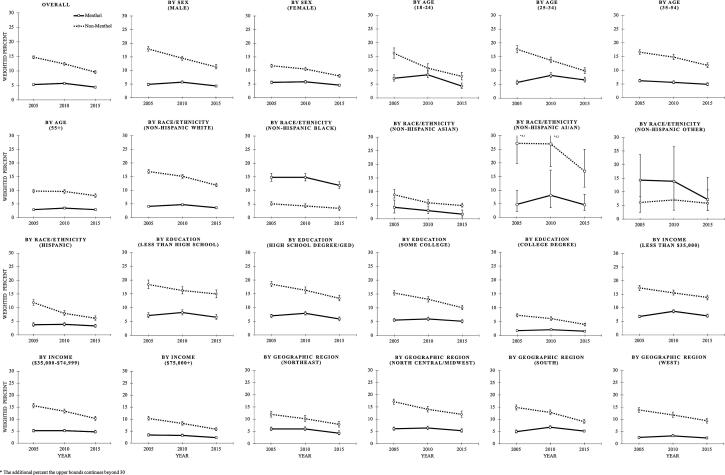
Trends in Prevalence of Menthol and Non-Menthol Cigarette Use by Sociodemographic and Geographic Characteristics, 2005–2015.

Table 2 presents changes in prevalence of menthol and non-menthol cigarette use from 2005 to 2010, 2010–2015, and 2005–2015. The prevalence of non-menthol cigarette use decreased by 15.5% between 2005 and 2010, and by an additional 22.5% between 2010 and 2015, for a net decrease of 34.6% from 2005 to 2015. The prevalence of menthol cigarette use increased by 9.0% between 2005 and 2010, but decreased by 22.9% between 2010 and 2015, for a net decrease of 15.9% from 2005 to 2015. From 2005 to 2015, the prevalence of non-menthol cigarette use decreased for all sociodemographic subgroups, although the decrease was not statistically significant for NH AI/ANs (p = 0.059) and NH Others (p = 0.132), possibly due to small sample size. In contrast, changes between 2005 and 2015 were more variable for menthol cigarette use. Generally, menthol cigarette use increased from 2005 to 2010 and decreased from 2010 to 2015. For example, among men, the prevalence of menthol cigarette use went from 4.9% (2005) to 5.7% (2010) to 4.3% (2015). The prevalence decreased over the entire time period overall and among females; participants aged 18–24 and 35–54 years old; NH Whites, NH Blacks, and NH Asians; participants with a high school degree or GED; participants with a family income of $75,000 or higher; and participants residing in the Northeast. However, menthol cigarette use remained stable or did not significantly decrease among males; adults aged 25–34 and 55 years and older; NH AI/ANs, NH Others, and Hispanics; participants with less than high school degree, some college, or a college degree; participants with a family income below $35,000 or between $35,000 and $74,999; and participants residing in the North Central/Midwest, South, and West over the entire time period. Fig. 1 depicts trends in menthol and non-menthol cigarette use overall and by sociodemographic and geographic characteristics.

**Table 2:** Changes in Prevalence of Current Menthol and Non-Menthol Cigarette Use by Sociodemographic and Geographic Characteristics, 2005–2015

Characteristics	Menthol						Non-Menthol					
	Year			% change			Year			% change		
	2005	2010	2015	2005–2010	2010–2015	2005–2015	2005	2010	2015	2005–2010	2010–2015	2005–2015
Overall	5.3	5.7	4.4	9.0%*	−22.9%**	−15.9%**	14.7	12.4	9.6	−15.5%**	−22.5%**	−34.6%**
Sex												
Male	4.9	5.7	4.3	17.5%*	−25.4%**	−12.3%	17.9	14.5	11.3	−19.3%**	−21.6%**	−36.7%**
Female	5.6	5.8	4.6	2.3%	−20.6%**	−18.8%**	11.7	10.5	8.0	−10.4%*	−23.6%**	−31.6%**

Age group												
18–24	7.1	8.4	4.3	18.0%	−49.3%**	−40.2%**	16.2	10.8	7.8	−32.9%**	−28.2%*	−51.8%**
25–34	5.7	8.2	6.6	45.6%**	−19.2%*	17.6%	17.7	13.7	9.9	−22.6%**	−27.9%**	−44.2%**
35–54	6.2	5.6	4.9	−10.7%	−11.9%	−21.3%*	16.6	14.8	11.8	−10.8%*	−20.1%**	−28.8%**
55+	2.9	3.5	2.9	18.4%	−16.0%*	−0.5%	9.7	9.6	8.0	−1.5%	−16.5%*	−17.7%**

Race/ethnicity												
Non-Hispanic White	4.1	4.7	3.6	14.8%*	−24.1%**	−12.8%*	16.9	15.1	11.9	−11.0%**	−20.8%**	−29.5%**
Non-Hispanic Black	14.8	14.8	11.9	−0.2%	−19.8%*	−20.0%*	5.1	4.3	3.4	−16.9%*	−19.6%	−33.2%*
Non-Hispanic Asian	4.2	3.0	1.7	−28.0%	−43.7%*	−59.5%*	8.8	5.9	4.9	−32.6%*	−17.4%	−44.3%*
Non-Hispanic AI/AN	4.8	8.1	4.7	68.3%	−41.4%	−1.4%	27.2	27.0	17.0	−0.6%	−37.2%	−37.6%
Non-Hispanic Other	14.3	13.9	7.2	−3.1%	−48.2%	−49.9%	6.1	7.0	5.8	14.9%	−17.0%	−4.6%
Hispanic	3.7	3.9	3.3	6.8%	−15.8%	−10.1%	11.9	7.9	6.1	−33.3%**	−23.0%*	−48.6%**

Education												
Less then high school	7.2	8.3	6.6	15.0%	−19.9%*	−7.9%	18.4	16.2	15.0	−11.6%*	−7.9%	−18.6%*
High school degree/GED	7.0	7.9	5.9	13.0%	−25.3%**	−15.6%*	18.5	16.3	13.4	−12.1%*	−17.9%**	−27.8%**
Some college	5.6	6.0	5.2	6.5%	−13.5%	−7.9%	15.4	13.1	10.1	−14.9%*	−22.9%**	−34.4%**
College degree	1.8	2.1	1.6	19.0%	−25.6%*	−11.5%	7.3	6.1	4.0	−16.8%*	−34.1%**	−45.1%**

Family income												
Less than $35,000	6.8	8.7	7.0	28.4%**	−19.5%**	3.5%	17.3	15.5	13.8	−10.0%*	−11.3%*	−20.1%**
$35,000-$74,999	5.2	5.2	4.6	−0.2%	−10.8%	−11.0%	15.6	13.3	10.3	−14.5%**	−22.5%**	−33.7%**
$75,000+	3.4	3.2	2.3	−5.0%	−29.7%*	−33.2%*	10.3	8.2	5.8	−20.3%**	−29.6%**	−43.9%**

Geographic region												
Northeast	6.0	6.0	4.3	1.0%	−28.1%*	−27.4%*	12.0	10.2	7.9	−14.8%*	−22.7%*	−34.2%**
North central/midwest	6.1	6.4	5.3	5.1%	−16.6%*	−12.3%	17.2	14.0	12.0	−18.3%**	−14.5%*	−30.1%**
South	5.9	6.8	5.2	15.7%*	−23.8%**	−11.8%	14.9	12.8	9.1	−13.8%*	−28.9%**	−38.7%**
West	2.6	3.2	2.4	26.0%	−27.0%*	−8.0%	13.8	11.8	9.4	−14.2%*	−20.6%*	−31.8%**

Some percent changes may not accurately reflect differences in proportions due to rounding.

Non-classifiable current cigarette users are excluded; sexual orientation is excluded due to having an estimate at one time point (2015). NHIS 2005 sample size = 31,132; NHIS 2010 sample size = 26,967; NHIS 2015 sample size = 33,541.

* *p* < 0.05; ** *p* < 0.001.

**Table A:** Prevalence of Current Cigarette Use by Sociodemographic and Geographic Characteristics, 2005–2015.

Characteristics	Cigarette Use					
	*n*	2005% (95% CI)	*n*	2010% (95% CI)	*n*	2015% (95% CI)
Overall	6511	20.9 (20.3, 21.5)	5147	19.3 (18.8, 19.9)	5415	15.1 (14.6, 15.7)
Sex						
Male	3324	23.9 (22.9, 24.9)	2613	21.5 (20.7, 22.4)	2749	16.7 (15.9, 17.6)
Female	3187	18.1 (17.5, 18.9)	2534	17.3 (16.6, 18.1)	2666	13.6 (12.9, 14.3)

Age group						
18-24	759	24.4 (22.4, 26.5)	547	20.1 (18.3, 22.0)	417	13.0 (11.4, 14.8)
25-34	1373	24.6 (23.1, 26.1)	1105	23.3 (21.9, 24.8)	1080	18.1 (16.8, 19.5)
35-54	2918	24.0 (23.1, 24.9)	2132	22.0 (21.0, 23.1)	2118	17.9 (16.9, 18.9)
55+	1461	13.2 (12.5, 13.9)	1363	13.8 (13.0, 14.6)	1800	11.7 (11.0, 12.5)

Race/ethnicity						
Non-Hispanic White	4461	22.0 (21.2, 22.8)	3298	21.1 (20.3, 21.8)	3658	16.6 (15.8, 17.4)
Non-Hispanic Black	936	21.4 (19.8, 23.0)	937	20.5 (19.1, 22.1)	814	16.8 (15.4, 18.4)
Non-Hispanic Asian	126	13.5 (10.8, 16.7)	178	9.4 (7.8, 11.2)	165	7.2 (5.8, 8.7)
Non-Hispanic AI/AN	52	32.6 (24.7, 41.6)	52	35.6 (27.0, 45.3)	93	24.2 (18.7, 30.6)
Non-Hispanic Other	15	20.4 (13.2, 30.2)	22	23.5 (15.2, 34.5)	32	13.2 (8.2, 20.6)
Hispanic	921	16.2 (15.1, 17.4)	660	12.5 (11.4, 13.6)	653	10.1 (9.1, 11.1)

Sexual orientation						
Straight/heterosexual	--	--	--	--	4993	14.9 (14.4, 15.5)
Lesbian/gay/bisexual	--	--	--	--	192	20.6 (17.1, 24.6)

Education						
Less then high school	1381	26.5 (24.8, 28.2)	1065	26.3 (24.8, 27.8)	1032	23.3 (21.6, 25.1)
High school degree/GED	2294	26.8 (25.7, 27.9)	1756	26.1 (24.8, 27.4)	1825	20.8 (19.6, 22.1)
Some college	1961	22.0 (21.0, 23.0)	1660	20.2 (19.1, 21.5)	1859	16.4 (15.4, 17.4)
College degree	821	9.6 (8.9, 10.3)	648	8.5 (7.8, 9.3)	673	6.0 (5.4, 6.6)

Family income						
Less than $35,000	3576	25.2 (24.2, 26.3)	2844	26.0 (24.9, 27.1)	3004	22.5 (21.5, 23.5)
$35,000-$74,999	1985	21.6 (20.6, 22.7)	1528	19.6 (18.6, 20.6)	1558	16.2 (15.2, 17.2)
$75,000+	950	14.5 (13.5, 15.5)	775	12.2 (11.4, 13.1)	853	8.6 (7.8, 9.4)

Geographic region						
Northeast	1075	19.2 (17.8, 20.7)	762	17.4 (16.2, 18.6)	813	13.5 (12.2, 14.9)
North central/midwest	1754	24.2 (23.0, 25.4)	1316	21.8 (20.4, 23.2)	1306	18.7 (17.4, 20.1)
South	2450	21.8 (20.6, 23.0)	2004	21.0 (20.0, 22.0)	1937	15.3 (14.5, 16.3)
West	1232	17.0 (16.0, 18.0)	1065	15.9 (14.7, 17.1)	1359	12.4 (11.4, 13.5)

628 participants were missing values for education.

1,397 participants were missing values for sexual orientation in NHIS 2015.

Sources: National Health Interview Survey, 2005, 2010, and 2015.

NHIS 2005 sample size = 31,132; NHIS 2010 sample size = 26,967; NHIS 2015 sample size = 33,541.

Prevalence estimates include non-classifiable menthol/non-menthol cigarette smokers.

## 4 Discussion

Our study provides nationally representative prevalence estimates of menthol and non-menthol cigarette use from 2005 to 2015 overall and by sociodemographic and geographic characteristics to give insight into trends in smoking disparities. Although the prevalence of non-menthol cigarette use decreased in all subgroups from 2005 to 2015, menthol cigarette use remained constant or did not significantly decrease among males; participants aged 25–34 and 55 years or older; Hispanics; participants with less than a high school degree, some college education, and a college degree; participants with a family income less than $75,000; and participants residing in the North Central/Midwest, South, and West.

Our findings from 2005 to 2010 are consistent with two NSDUH studies reporting menthol cigarette use has either remained stable or increased in certain sociodemographic groups (Giovino et al., 2015, Villanti et al., 2016). Menthol cigarette use in NSDUH increased from 2004 to 2010 (Giovino et al., 2015) and from 2008 to 2014 (Villanti et al., 2016) in participants aged 18–25. We reported similar increases in menthol cigarette use in participants aged 18–24 from 2005 to 2010, but not 2005 to 2015. Like Villanti et al, we observed an increase in menthol cigarette use for respondents aged 25–34 from 2005 to 2015, although our finding was not statistically significant (p = 0.099). Further, our results indicated that menthol cigarette use did not change for NH Blacks from 2005 to 2010, consistent with an older study using NSDUH data from 2004 to 2010 (Giovino et al., 2015). Our declining estimates from 2010 to 2015 and 2005 to 2015 for NH Blacks differ from NSDUH data from 2008 to 2014 (Villanti et al., 2016), which suggested an unchanging prevalence. However, a more recent examination reported a reduction in menthol cigarette use prevalence among NH Blacks from 2002 to 2016, consistent with our findings (Weinberger et al., 2019). Variability of results across studies may be due to differences in analytic strategies used to evaluate prevalence in menthol cigarette use over time, including what years were selected for prevalence estimate comparisons, and the smoking variable definitions (i.e., current cigarette use and brand use). For example, in NSDUH participants were asked if the cigarettes they smoked during the past 30 days were menthol, which differs from the brand preference assessment in NHIS. Nevertheless, our results suggest that certain groups are more susceptible to the long-term health consequences of menthol cigarette use.

Cigarette manufacturers have promoted mentholated products as healthier alternatives among targeted populations such as young smokers, women, and African Americans (U.S. Department of Health and Human Services, 2014, U.S. National Cancer Institute, , 2017). In 2009, the Tobacco Control Act granted the Food and Drug Administration (FDA) regulatory authority over the tobacco industry, including product flavoring. The FDA subsequently banned all cigarette flavors apart from menthol. This regulation could potentially explain why the prevalence of menthol cigarette use increased from 2005 to 2010, as smokers who used cigarettes with flavorings other than menthol may have switched to menthol flavoring after the 2009 ban. The overall decrease in menthol use between 2010 and 2015 may be due to the result of successful cessation efforts but could also reflect increased use of other flavored nicotine products (e.g., e-cigarettes, little cigars, cigarillos) during this time period (Kuiper et al., 2017, McMillen et al., 2014).

In 2018, the FDA introduced a plan to ban menthol cigarettes (Schroth et al., 2019). Since then, scholars have reviewed the implications of a menthol ban, including how the tobacco industry may retaliate (Schroth et al., 2019). Nevertheless, adult menthol cigarette users believe such a ban may help motivate them to quit smoking (Wackowski et al., 2014), and simulation models have depicted the potential to reduce deaths at the population level (Levy et al., 2011). Our results suggest that menthol cigarette users who belong to certain sociodemographic and geographic subgroups, such as young adults, racial/ethnic minorities, and people residing in the Northeast, North Central/Midwest, and South, may benefit more from a ban. Additionally, our results emphasize urgency in addressing menthol cigarette use among sexual minority adults (Fallin et al., 2015).

This study comes with several limitations. First, the NHIS Cancer Control Supplements occurred in five-year intervals, preventing us from conducting a more refined analysis of the time period, or from examining trends after 2015. Moreover, the cross-sectional nature precludes us from determining changes in menthol cigarette use at the individual level. Our study also relies on self-reported usual brand to identify menthol smokers. Such assessment fails to distinguish between participants who predominantly use menthol cigarettes and participants who may be more ambivalent about their brand preference. Furthermore, small sample sizes for certain sociodemographic subgroups might have limited our ability to observe significant differences over time. Despite these limitations, this study updates the current literature on sociodemographic and geographic trends in menthol cigarette use using the NHIS.

## 5 Conclusion

Our study reveals that the prevalence of non-menthol cigarette use has decreased from 2005 to 2015, while the prevalence of menthol cigarette use remained relatively constant across multiple sociodemographic and geographic subgroups. Given that 40% of cigarette users prefer menthol brands (Villanti et al., 2016), FDA endorsement of limitations on menthol cigarette manufacturing may improve public health, especially among populations that are disproportionately targeted by tobacco manufacturers and continue to smoke menthol cigarettes.

Research reported in this publication was supported by the National Cancer Institute of the National Institutes of Health (NIH) and FDA Center for Tobacco Products (CTP) under Award Number U54CA229974. The content is solely the responsibility of the authors and does not necessarily represent the official views of the NIH or the Food and Drug Administration.

## CRediT authorship contribution statement

**Delvon T. Mattingly:** Conceptualization, Methodology, Data curation, Formal analysis, Writing - original draft. **Jana L. Hirschtick:** Conceptualization, Methodology, Data curation, Formal analysis, Supervision, Writing - review & editing. **Rafael Meza:** Conceptualization, Funding acquisition, Writing - review & editing. **Nancy L. Fleischer:** Conceptualization, Funding acquisition, Writing - review & editing.

## Declaration of competing interest

The authors declare that they have no known competing financial interests or personal relationships that could have appeared to influence the work reported in this paper.
